# Mathematical Distinction in Action Potential between Primo-Vessels and Smooth Muscle

**DOI:** 10.1155/2012/269397

**Published:** 2012-01-29

**Authors:** Seong-Jin Cho, Sang-Hun Lee, Wenji Zhang, Sae-Bhom Lee, Kwang-Ho Choi, Sun-Mi Choi, Yeon-Hee Ryu

**Affiliations:** Acupuncture, Moxibustion & Meridian Research Center, Division of Standard Research, Korea Institute of Oriental Medicine, Daejeon 305-811, Republic of Korea

## Abstract

We studied the action potential of Primo-vessels in rats to determine the electrophysiological characteristics of these structures. We introduced a mathematical analysis method, a normalized Fourier transform that displays the sine and cosine components separately, to compare the action potentials of Primo-vessels with those for the smooth muscle. We found that Primo-vessels generated two types of action potential pulses that differed from those of smooth muscle: (1) Type I pulse had rapid depolarizing and repolarizing phases, and (2) Type II pulse had a rapid depolarizing phase and a gradually slowing repolarizing phase.

## 1. Introduction

Acupuncture has been an important part of oriental medicine for thousands of years. However, due to the lack of anatomical study of meridians and the lack of scientific proof of the existence of Qi, the nature of oriental medicine is controversial. Many researchers have studied meridians, acupuncture points, and Qi circulation [1–10]. However, the results of these studies have also been disputed because they were limited in topology and/or have not been repeated often. Kim published his findings on the substance of meridians in the early 1960s [11, 12]. He reported that meridians made up a new system in the living body, different from both the nervous system and the blood or lymphatic vessels. Unfortunately, he did not disclose the staining materials or methods used to observe the claimed structures; therefore, interest in such studies has declined over the past 40 years. Recently, certain researchers have become interested in Primo-vascular system (the scientific name for Bonghan system) and have tried to investigate Primo-vascular system by finding Primo-node (Bonghan corpuscle) and Primo-vessels (Bonghan ducts) [13–18]. It was too difficult to study the functions of Primo-vessels directly because of their small size. Therefore, more research based on electrophysiology was needed, for example, action potential measurements. It is well known that tissues have different action potentials depending on their structure and function. Thus, the function of a tissue can be inferred by analyzing its action potential. The study presented herein was a comparison of the action potentials generated by Primo-vessels with the pacemaker potential, made by smooth muscle from the small intestine. We also introduced a mathematical analysis method, a normalized Fourier transform that is more useful for viewing the waveform and phase than the power spectrum from a general Fourier transform.

## 2. Materials and Methods

### 2.1. Animals and Tissue Preparation

Male 7-week-old Sprague-Dawley rats weighing 250–320 g were used. The rats were anesthetized with an injection of 1.5 g/kg urethane (C_3_H_7_NO_2_) into the femoral region, and the entire surgical procedure was performed with the rat in the anesthetized state. A midline abdominal incision was made, and the internal organs were exposed. Smooth muscle from the small intestine and the small intestine surface Primo-vessels were removed from the rats and placed on Sylgard. Primo-vessels were identified by its anatomical characteristics described in Kim's study [12]. And we dropped phosphate buffered saline (PBS, contained (in mM) 137 NaCl, 27 KCl, 10 Na_2_HPO_4_, 2 KH_2_PO_4_) on the sample.

### 2.2. Equipment

Tissue preparation was viewed under a microscope (SMZ1500, Nikon, Japan). Light was supplied by a Fiber-Lite (MI-150, Dolan Jenner Industries, MA). Another microscope (SZ61, Olympus, Japan) was used for insertion of electrode (0.002′′ bare tungsten wire, A-M Systems, WA) to tissue. Data were acquired by a data acquisition system (PowerLab/16SP, ADInstruments, CO) and amplified by Bio Amp (ML131, ADInstruments, CO). Data acquisition program (LabChart 6, ADInstruments, CO) was used to record. Data were analyzed with Microsoft Excel 2010 (Microsoft, WA).

### 2.3. Extracellular Recording

An electrode was placed in the tissue on Sylgard. The tissue was perfused with Kreb's solution at a constant flow rate of about 5 mL/min. Kreb's solution contained (in mM) 10.10 D-glucose, 115.48 NaCl, 21.90 NaHCO_3_, 4.61 KCl, 1.14 NaH_2_PO_4_, 2.50 CaCl_2_, and 1.16 MgSO_4_. This solution had pH 7.4 at 36°C. The temperature of the solution in the organ bath was maintained at 36~38°C. Electrical responses were amplified, low pass filtered (50 Hz), and recorded (time interval: 0.0005 sec) on a computer. The laboratory was isolated from electromagnetic noise by Faraday cage.

### 2.4. Analysis

The whole action potential pulses recorded from the smooth muscles and Primo-vessels were each extracted separately. All pulses were differentiated by time. For normalizing, the depolarizing and repolarizing sections of each pulse were selected. Amplitude and time were rescaled to have value from 0 to 1; this ensured that every pulse had the same size on the *x*-axis and *y*-axis for a comparison of only the pulse shape. To distinguish the waveform, normalized pulses were Fourier transformed [19], and the coefficients for the sine and cosine components were displayed separately. The coefficients for the sine components were dotted on the negative *x*-axis and those for the cosine components were dotted on the positive *x*-axis. The coefficients were derived using a Fourier transform, as shown in:

(1) $$\begin{array}{cc} A\left(f\right) & =\overset{1}{\underset{0}{\int }}x\left(t\right)[\left\{1-\mathrm{sign}\left(f\right)\right\}\sin\left(2\pi ft\right)\\ & \;\;\;\;\;\;+\left\{1+\mathrm{sign}\left(f\right)\right\}\cos\left(2\pi ft\right)]dt. \end{array}$$

The following is the inverse normalized Fourier transform:

(2) $$\begin{array}{cc} x\left(t\right) & =\frac{A\left(0\right)}{2}\\ & \;+\overset{\infty }{\underset{-\infty }{\sum }}A\left(f\right)\{\frac{1-\mathrm{sign}\left(f\right)}{2}\sin\left(2\pi ft\right)\\ & \;\;\;\;\;+\frac{1+\mathrm{sign}\left(f\right)}{2}\cos\left(2\pi ft\right)\},\\ & \;\;\;\;\;f:\text{integer},\;\mathrm{sign}\left(f\right):\begin{array}{cc} -1: & f<0\\ 0: & f=0\\ 1: & f>0. \end{array} \end{array}$$

In this study, the Fourier transform was performed in a 0–10 frequency range. Additionally, the amplitude, the FW (full width, the time from depolarization back to repolarization at the rest potential), and *t*_max⁡_ (the time from the rest potential to the maximum potential) were calculated.

## 3. Results

**Figure 1:**
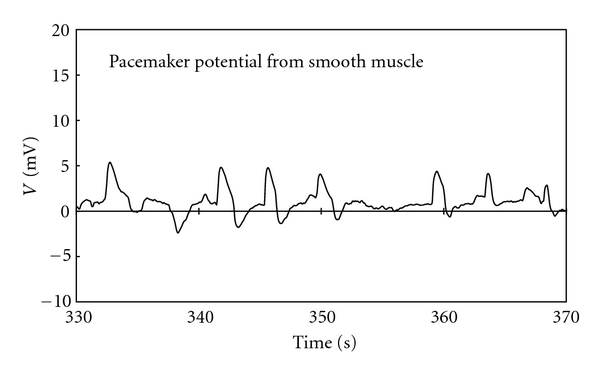
The pacemaker potential waves from smooth muscle in small intestine. The pulses rose fast and decreased gradually before falling to the rest potential. The pulses were generated periodically and had uniform amplitude.

We identified action potential waves in the smooth muscle called the pacemaker potentials. These waves had a rapid rising phase followed by a plateau component with monotonically declining amplitude.  Figure 1 shows some of the pacemaker pulses from the smooth muscle. These pulses were generated periodically having frequency of 17.7 ± 5.0/min. Also, there is uniform amplitude in these pulses [20–24]. 

**Figure 2:**
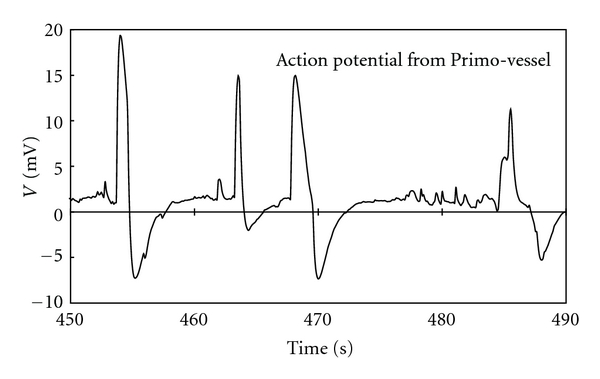
The action potential waves from Primo-vessel. The pulses rose fast and fell to the rest potential immediately. The pulses were generated aperiodically having irregular amplitude. The amplitude of these pulses was larger than those of smooth muscle.

On the other hand, different types of potential waves were found in Primo-vessels.  Figure 2 shows some of the action potential waves from Primo-vessels. These pulses differed from those of smooth muscle in their fast rise and fall as well as their larger amplitude. The pulses were generated aperiodically and amplitude was irregular. 

**Figure 3:**
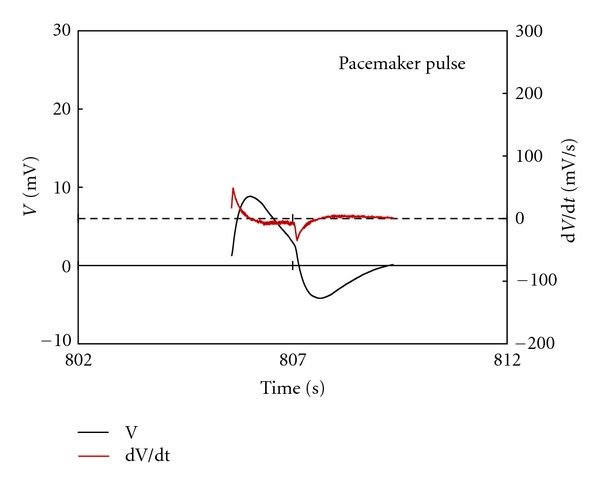
A representative action potential pulse from smooth muscle in small intestine and its derivation. The derivative had a shape with short, sharp positive and long, flat negative within depolarizing from the rest potential and repolarizing to the rest potential.

Ninety-one pulses from smooth muscle and 180 pulses from the Primo-vessels were extracted.  Figure 3 shows a representative pacemaker pulse and its derivative. The mean amplitude of these pulses was 4.45 ± 3.02 mV, and it took 0.39 ± 0.16 s to rise to the maximum potential and 1.83 ± 0.90 s to return to the rest potential (Table 1). The derivative had a shape with short, sharp positive and long, flat negative. There were some different two types of pulse in records from Primo-vessels.

**Figure 4:**
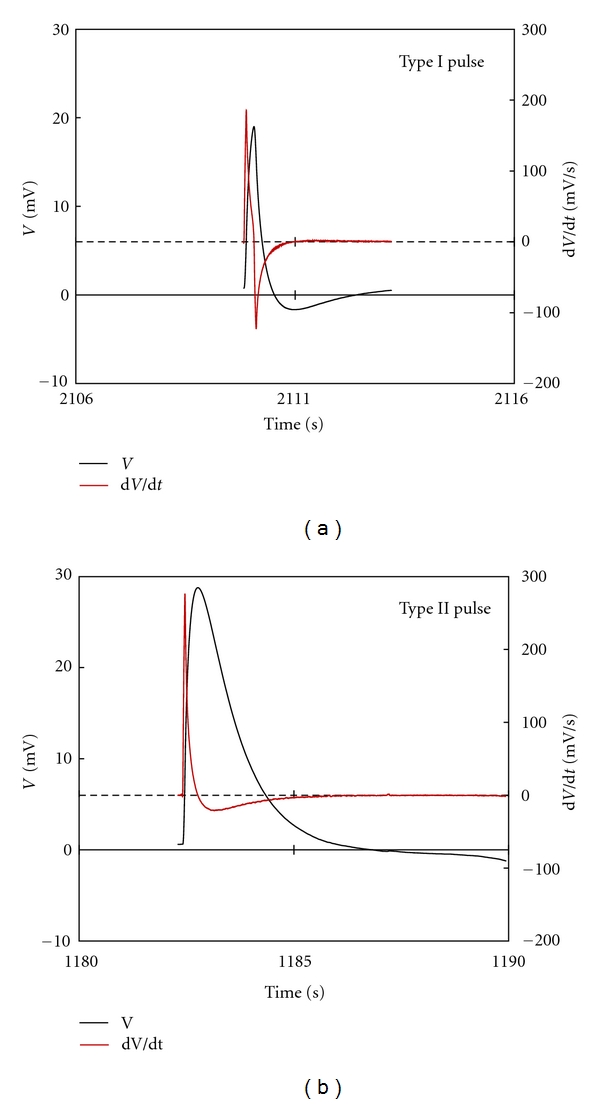
Representative traces for the two types of action potential pulses from Primo-vessels and their derivatives. The derivative of Type I pulse had a shape with short, sharp positive and negative within depolarizing from the rest potential and repolarizing to the rest potential. For the Type II, the derivative consisted of short, sharp positive and broad, hill-like negative. There was no significant difference in amplitude of these two types, but huge difference existed in FW.

Figure 4 shows representative pulses of the two types generated in Primo-vessels. Type I pulses had mean amplitude of 10.02 ± 8.36 mV, and it took 0.31 ± 0.10 s to rise to the maximum potential and 0.88 ± 0.38 s to return to the rest potential. The derivative of this type had a shape with short, sharp positive and negative. Type II pulses had mean amplitude of 13.67 ± 8.69 mV, and it took 0.38 ± 0.15 s to rise to a maximum potential and 2.75 ± 1.17 s to return to the rest potential. The derivative of this type had a shape with short, sharp positive and broad, hill-like negative. This shape was similar to the shape of pacemaker, but both of the two patterns were clearly different. The derivative of pacemaker pulse had flat and not decreasing negative value; however, that of Type II did not have flat but decreasing negative value.

**Figure 5:**
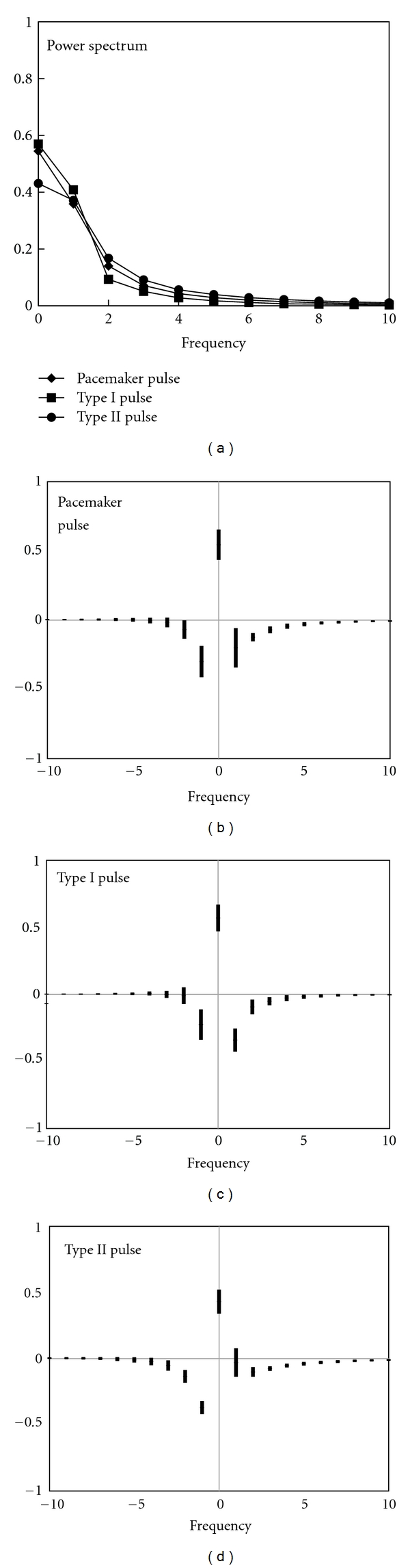
The coefficients from normalized Fourier transformed pulses. Sine components are displayed on the negative *x*-axis, and cosine components are displayed on the positive *x*-axis. The bars on the graph are the mean distribution of the dots. Frequency has arbitrary unit. (a) Power spectrum could not distinguish each type of pulse. (b–d) The normalized Fourier transform and separate displaying of coefficients could distinguish each type of pulse. Pacemaker pulse had two dominant components with a slight superiority of the sine component. In contrast, the cosine component was slightly larger than the sine component in Type I pulse. Type II pulse had only one dominant sine component.

 For the detailed comparison of the shape of the action potential pulses from smooth muscle and Primo-vessels, all pulses were normalized and Fourier transformed at the frequency range of 0 to 10.  Figure 5 shows the results of a normalized Fourier transform for the pulses from smooth muscle and for the two types of pulses from Primo-vessels. For the result from smooth muscle, the first sine and cosine components were dominant with a slight superiority of the sine component. The Type I pulses had the same dominant components, but the cosine component was slightly larger than the sine component. On the other hand, for the result of the Type II pulses, only the first sine component was dominant. Power spectrum of three types of pulses had same pattern and values.

## 4. Discussion

In this study, we investigated the mathematical difference between the action potentials of the smooth muscle and Primo-vessels. In the results from the smooth muscle, we found the periodic pulses with uniform amplitude. This result is similar to that of the study by Kito and Suzuki [20]. This similarity demonstrates that extracellular recording is reliable and repeatable. Though extracellular recording has inaccuracy in amplitude and rest potential, it supports enough information to study about periodic pattern and waveform of action potential. Therefore, we studied the action potential of Primo-vessels by extracellular recording to determine their electrophysiological characteristics. Based on the Primo-vessel pulse results, it seemed that there was no severe periodic repetition and that the amplitude had a large variation. This result indicates that Primo-vessels may not act periodically and that the intensity of the action is not uniform. Aperiodic pattern and varying amplitude can be shown also in the study by Choi et al. [23]. In addition, the derivatives of the pulses for smooth muscle and Primo-vessels had different patterns in between. The pulses from the smooth muscle had rapid depolarization phase and consistently slow repolarization phase. However, the pulses from the Primo-vessels had two types of derivative patterns. Type I pattern demonstrated that depolarization and repolarization were fast. Type II pattern signified that depolarization is rapid and that repolarization started rapidly but slowed gradually. These phenomena are shown by the FW and *t*_max⁡_ values. The *t*_max⁡_ for the three types were short suggesting that depolarization was fast. The FW values varied greatly, indicating that the repolarization velocities were different for each type of pulse. It suggests that Primo-vessels have at least two types of cells generating action potential.

For a more analytical comparison, all pulses were normalized and Fourier transformed. The dominant coefficient distribution implies that frequency density was maximal at 1 in the three pulses, although their phases were different. With respect to the characteristics of the sine and cosine waves, the maximum amplitude of the sine wave leaned toward the side, but the maximum amplitude of the cosine wave was located in the center. For the pulses from the smooth muscle, the sine component was slightly larger than the cosine component. In contrast, for the Type I pulse, the cosine component was slightly larger than the sine component. These observations indicate that the pulses from the smooth muscle had an asymmetrical structure that leaned toward the left and that the Type I pulses had a more symmetrical structure. For the pacemaker pulse, faster depolarization than repolarization causes the pulse to lean toward the left, and a slow repolarization pulse maintains a potential higher than half of the amplitude in the middle of the FW. On the other hand, the symmetry of the fast depolarization and repolarization generates a dominant cosine component, and the slight lean toward the left and the asymmetry of the curvature creates a dominant sine component that is slightly smaller in Type I pulse. However, only the first sine component was dominant for the Type II pulses. The dominance of the sine component implies that depolarization is faster than repolarization, as for smooth muscle. The lack of a cosine component suggests that repolarization started rapidly, and thus the potential went down to under half of the amplitude in the middle of the FW, and then the repolarization slowed gradually. The larger second sine component in Type II pulses indicates that the pulses leaned more toward the left. We also compared power spectrum of three types of pulses. Power spectrum of three types of pulses had same pattern and values. Judging from the results of power spectrum, it was identified that the action potentials of smooth muscle and Primo-vessels were not distinguished by power spectrum since power spectrum provided fragmentary information, only frequency density. Therefore, the normalized Fourier transform serves as a more sophisticated criterion for pulse distinction in comparison with power spectrum.

## 5. Conclusion

We found that Primo-vessels generated different types of the action potentials from smooth muscle located nearby using a simple measurement of the FW, the pulse derivatives, and a normalized Fourier transform. There were two types of pulses generated by the Primo-vessels: Type I pulses had fast depolarizing and repolarizing phases, and Type II pulses had a fast depolarizing phase and gradually slowing repolarizing phase. The sharp top and larger amplitude of the pulses generated by Primo-vessels could distinguish them from the pulses generated by smooth muscle; thus, it is possible to assume that Primo-vessels perform a different function from the smooth muscle. For confirmation of this hypothesis, further study is needed regarding the physiological mechanism responsible for generating these pulses and regarding which ion channels are used.

## Figures and Tables

**Table 1:** Amplitude, FW, and *t*_max⁡_ of the action potentials from smooth muscle and Primo-vessels. The amplitude means the difference between the maximum potential and the rest potential. The FW means the time from depolarizing to repolarizing to the rest potential. The *t*_max⁡_ means time that is taken to arrive at the maximum potential. The amplitude of the action potential from Primo-vessels was larger than that from smooth muscle. It took similar time for depolarizing of all types of pulses including pacemaker, but their repolarizing time varied.

	Amplitude (mV)	FW (s)	*t*_max⁡_ (s)
Pacemaker pulse	4.45 ± 3.02	1.83 ± 0.90	0.39 ± 0.16
Type I pulse	10.02 ± 8.36	0.88 ± 0.38	0.31 ± 0.10
Type II pulse	13.67 ± 8.69	2.75 ± 1.17	0.38 ± 0.15
